# Coupling GIS spatial analysis and Ensemble Niche Modelling to investigate climate change-related threats to the Sicilian pond turtle *Emys trinacris*, an endangered species from the Mediterranean

**DOI:** 10.7717/peerj.4969

**Published:** 2018-06-05

**Authors:** Mattia Iannella, Francesco Cerasoli, Paola D’Alessandro, Giulia Console, Maurizio Biondi

**Affiliations:** Department of Life, Health & Environmental Sciences, University of L’Aquila, L’Aquila, Italy

**Keywords:** Species distribution models, Ensemble forecast, *Emys trinacris*, Global warming, Protected Areas network, Gap analysis

## Abstract

The pond turtle *Emys trinacris* is an endangered endemic species of Sicily showing a fragmented distribution throughout the main island. In this study, we applied “Ensemble Niche Modelling”, combining more classical statistical techniques as Generalized Linear Models and Multivariate Adaptive Regression Splines with machine-learning approaches as Boosted Regression Trees and Maxent, to model the potential distribution of the species under current and future climatic conditions. Moreover, a “gap analysis” performed on both the species’ presence sites and the predictions from the Ensemble Models is proposed to integrate outputs from these models, in order to assess the conservation status of this threatened species in the context of biodiversity management. For this aim, four “Representative Concentration Pathways”, corresponding to different greenhouse gases emissions trajectories were considered to project the obtained models to both 2050 and 2070. Areas lost, gained or remaining stable for the target species in the projected models were calculated. *E. trinacris*’ potential distribution resulted to be significantly dependent upon precipitation-linked variables, mainly precipitation of wettest and coldest quarter. Future negative effects for the conservation of this species, because of more unstable precipitation patterns and extreme meteorological events, emerged from our analyses. Further, the sites currently inhabited by *E. trinacris* are, for more than a half, out of the Protected Areas network, highlighting an inadequate management of the species by the authorities responsible for its protection. Our results, therefore, suggest that in the next future the Sicilian pond turtle will need the utmost attention by the scientific community to avoid the imminent risk of extinction. Finally, the gap analysis performed in GIS environment resulted to be a very informative post-modeling technique, potentially applicable to the management of species at risk and to Protected Areas’ planning in many contexts.

## Introduction

The Sicilian pond turtle, *Emys trinacris,* was described by Fritz et al. (2005), who separated it from the European pond turtle *E. orbicularis* (Linnaeus) on the basis of genetic differences. Since then, some studies on morphology and genetics (D’Angelo, 2006; Fritz et al., 2006, 2007; D’Angelo, Galia & Lo Valvo, 2008; Spadola & Insacco, 2009; Pedall et al., 2011; Manfredi et al., 2013; Vamberger et al., 2015) have been published, contributing to a better characterization of the taxonomy and phylogeography of this species. On the other hand, we have not yet got a complete autoecological profile of *E. trinacris* (Turrisi, 2008; Di Cerbo, 2011), although several of its phenological and ecological traits were described (Naselli-Flores et al., 2007; D’Angelo, Galia & Lo Valvo, 2008; D’Angelo et al., 2013; Lo Valvo et al., 2014). These studies always carried out on a limited number of localities (Naselli-Flores et al., 2007; D’Angelo, Galia & Lo Valvo, 2008; D’Angelo et al., 2013), have not yet permitted to fill the considerable gaps in the actual comprehension of the species’ habitat requirements.

Such a shortage in the overall knowledge about *E. trinacris* has particular relevance with respect to its conservation status. This species, strictly endemic to Sicily Island, may be considered at risk of extinction for several reasons (Di Cerbo, 2011), including its low dispersal potential (Lo Valvo, D’Angelo & Regina, 2008; Lo Valvo et al., 2014), the reduction in the number of populations and the scarce gene flow (Turrisi, 2008). This species is also notably linked to aquatic environments, such as estuaries, wetlands and lentic habitats (Turrisi, 2008; Di Cerbo, 2011), which are all highly threatened because of recent land use modifications and ongoing and future climate change (Chang et al., 2015; Wu et al., 2017). The Mediterranean area, in fact, seems to be particularly sensitive to these modifications, which may bring to severe alterations in water balance (Millán et al., 2005; Somot et al., 2008; Garcia et al., 2017a, 2017b) and to a higher risk of extreme meteorological events (Romera et al., 2016). Markovic et al. (2017) showed that the Mediterranean islands have the most vulnerable freshwater ecosystems, with respect to future climate change, if compared with those of continental Europe. However, notwithstanding these threats, it is interesting to note that the current European protected area network covers less than one quarter of the overall extent of the most vulnerable freshwater catchments (Markovic et al., 2017). Moreover, in Sicily Island the protection of the territory is relatively young: the first protected areas (PAs) were established in the 1980s, with most of them created in the middle 1990s and the last ones created in the context of the Natura 2000 project.

Ecological Niche Models (ENMs) represent in this context a powerful methodological tool to investigate both the drivers shaping the current distribution of endangered species and the potential new threats related to climate change and land use modifications. Alongside with their applications to the different research fields of conservation biology (Araújo et al., 2011; Guisan et al., 2013; Ficetola et al., 2015; Cerasoli et al., 2017), ENMs have been intensively applied also to biogeography issues (Richards, Carstens & Lacey Knowles, 2007; Nogués-Bravo, 2009; Wielstra et al., 2013; Iannella, Cerasoli & Biondi, 2017), as well as to the hybrid discipline of conservation biogeography (Franklin, 2013). Moreover, notwithstanding most of papers applying ENMs to the above-cited research fields focus on large scale (i.e., continental to global) patterns (Araújo, Thuiller & Pearson, 2006; Ficetola, Thuiller & Miaud, 2007; Araújo et al., 2011; Ficetola et al., 2015; Stralberg et al., 2015), other researches based on the implementation of ENMs over national (Guisan & Hofer, 2003; Ferreira et al., 2013; Reino et al., 2017) and regional (Lyet et al., 2013; Cerasoli et al., 2017; Urbani et al., 2015; Urbani, D’Alessandro & Biondi, 2017) extents showed that these modeling techniques allow the opportunity to gain deep insights into the constraints imposed on species’ distributions by the availability of suitable environmental conditions at local scales.

In this contribution, we report the results of a research, carried out by means of ENM techniques, on the possible climatic variables affecting *E. trinacris*’ current and future distribution. Starting from models based on the current climatic conditions, we inferred possible modifications in the future distribution of the Sicilian pond turtle across four different global warming scenarios. Finally, a gap analysis in GIS environment was performed taking into consideration the protected area network in Sicily and the current and future potential distribution of *E. trinacris*.

## Materials and Methods

### Target species and study area

**Figure 1:**
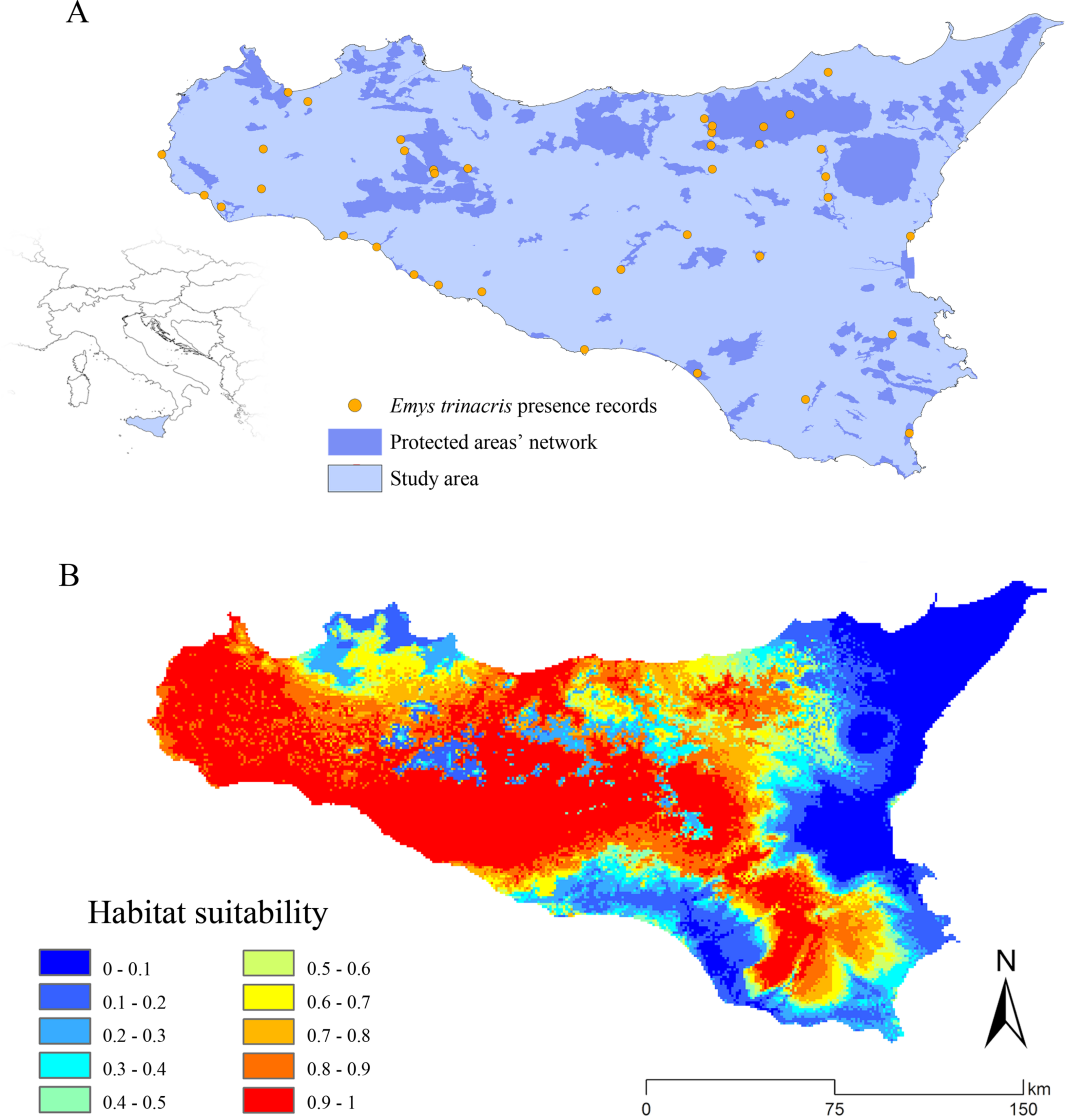
Study area and predicted habitat suitability for *Emys trinacris*. Study area and presence records of the target species, *Emys trinacris* (Fritz et al., 2005); (A) the Protected Areas’ network (both nationally- and internationally-established) of study area is highlighted in blue. (B) Map of habitat suitability, discretized in 10 classes, obtained from the Ensemble Modelling process performed over *Emys trinacris* presence and pseudo-absences records.

The target species of our analyses is the Sicilian pond turtle *E. trinacris* (Fritz et al., 2005), classified as “Endangered—A2c” within the Italian IUCN Red List (Rondinini et al., 2013) and “Data deficient” in the IUCN global database (van Dijk, 2009). This species is found throughout the whole Sicily, showing a wide but fragmented range. There is noticeably contradictory information between local (Turrisi, 2008), national (Di Cerbo, 2011) and international (van Dijk, 2009) bibliographic sources dealing with the distribution of this species. For our aims, we generated a database of 39 occurrence records (see Fig. 1A and Supplement 1), integrating GPS-precision literature data with unpublished observations in order to avoid the use of over-simplified centroids from 10 × 10 km cells of atlases in the modeling process. The study area comprehends only the main island, because the target species does not currently occur in the surrounding minor islands.

### Model building

The nineteen Worldclim (ver. 1.4) bioclimatic variables were chosen as candidate predictors (Hijmans et al., 2005), with 30 arc-seconds resolution, for both the current and the future scenarios (Supplement 2). We chose to use, for the model projections to 2050 and 2070, four different “Representative Concentration Pathways” (commonly known as RCPs) (Meinshausen et al., 2011; Stocker, 2014), coded as 2.6, 4.5, 6.0 and 8.5. The RCP 2.6 predicts a low future increase in radiative forcing with respect to its current values, consequent on a constant decrease in greenhouse gases (GHG) emissions after a predicted peak in 2020, while, at the other extreme, the RCP 8.5 corresponds to the highest radiative forcing increase, with a non-stop GHG emission trajectory until 2100 (Riahi et al., 2011); 4.5 and 6.0 are intermediate RCPs, representing gradually-increasing values of radiative forcing. Even though the RCP 2.6 is by now acknowledged to be no more plausible (Sanford et al., 2014), it was implemented in the ensemble modeling framework as a “control scenario”, providing information on how more efficient measures against global warming could have influenced the future distribution of the target species. Since the variability in the future climate conditions inferred by different Global Climate Models (GCMs) is recognized as one of the most important sources of uncertainty in ENMs projections to future scenarios (Garcia et al., 2012; Stralberg et al., 2015), we chose to perform model projections to 2050 and 2070 considering three different GCMs, namely BCC-CSM-1 (Wu et al., 2014), CCSM4 (Gent et al., 2011) and MIROC-ESM (Watanabe et al., 2011). Possible multicollinearity within the set of 19 candidate predictors was assessed through a correlation matrix (Supplement 3) built in ArcMap 10.0 (Esri, Redlands, CA, USA); within pairs of variables with Pearson |*r*| > 0.85, the one having less ecological importance to the species based on its autoecology (Di Cerbo, 2011; Lo Valvo et al., 2014) was discarded from model building (Elith et al., 2006; Dormann et al., 2013; Brandt et al., 2017). Occurrences were spatially rarefied using the spThin R package, setting the thinning distance to 10 km (Aiello-Lammens et al., 2015), and spatial autocorrelation among occurrence records was further tested through a Moran’s I test in ArcMap 10.0 (Esri, Redlands, CA, USA).

The “biomod2” package (Thuiller et al., 2016), implemented in R environment (R Core Team, 2016), was used to build the ENMs for *E. trinacris*. In particular, Ensemble Models (EMs, i.e., models resulting from the combination of individual ENMs obtained through different modeling algorithms) were built for the current climatic conditions through the “BIOMOD_EnsembleModeling” function, and then projected to the future scenarios by means of the “BIOMOD_EnsembleForecasting” function. Ten sets of 1,000 pseudo-absences each were generated through the Surface Range Envelope algorithm (Barbet-Massin et al., 2012; Reino et al., 2017), setting the quantile at 0.05 (i.e., pseudo-absences were randomly generated outside of the 95th quantile of the linear environmental envelope built on presence points): this strategy contributes to lower the probability of selecting pseudo-absences from suitable but uncolonized area, which would lead to increasing commission error (Brown & Yoder, 2015), and is considered fair when the aim of the study is not to model the realized distribution of a species but to investigate the potential one (Chefaoui & Lobo, 2008; Jiménez-Valverde, Lobo & Hortal, 2008), as in our case.

Models built for *E. trinacris* were parametrized as follows: Generalized Linear Models (GLM): type = “quadratic”, interaction level = 3; Multiple Adaptive Regression Splines (MARS): type = “quadratic”, interaction level = 3; Generalized Boosting Model, also known as Boosted Regression Trees (BRT): number of trees = 5,000, interaction depth = 3, cross-validation folds = 10; maxent (MAXENT.Phillips): maximum iterations = 5,000, betamultiplier = 2 (in order to obtain smoother model responses, Elith et al. (2011)). The choice of these techniques permitted to explore responses from different classes of models, ranging from more classical statistical techniques (GLMs) to machine learning-oriented approaches (BRT and Maxent). GLMs and MARS are based on parametric and piecewise linear functions, respectively (Leathwick et al., 2005; Elith et al., 2006); we set for both algorithms the type parameter to “quadratic” to produce smoother response functions and lower the risk of extreme extrapolation, with respect to polynomial formula, when projecting to future climate beyond the limits of current climate conditions on which models were calibrated. BRT combines the regression-tree and boosting algorithms to optimize predictive performance from an ensemble of trees sequentially fitted focusing on residuals from the previous iterations (Elith, Leathwick & Hastie, 2008); this technique has been shown to be good at selecting relevant variables and model interactions among them, and it generally results in high discrimination performance and fit of accurate functions (Elith et al., 2006; Elith, Leathwick & Hastie, 2008; Elith & Graham, 2009; Cerasoli et al., 2017), even though some overfitting problems were also shown, especially when data do not extensively cover the available environmental space (Elith & Graham, 2009; Cerasoli et al., 2017). Maxent, instead, represents a pure machine learning technique searching for the distribution of maximum entropy conditional to constraints on the difference between the expected values of the predictors under such distribution and their observed values (Elith et al., 2006; Phillips, Anderson & Schapire, 2006); even though it has often been acknowledged as one of the best performing modeling algorithms (Elith et al., 2006; Pearson et al., 2007), recent studies showed that its outputs and performance strongly depend on the chosen parameterization (Merow, Smith & Silander, 2013; Radosavljevic & Anderson, 2014). Finally, the Ensemble Modelling approach we implemented, based on weighted-averaging of the single ENMs’ predictions (see below), allows to obtain robust predictions (Araujo & New, 2007; Marmion et al., 2009) combining the strengths of the single algorithms and mitigating the respective weaknesses.

### Model evaluation and ensemble forecast

Discrimination performance of the single models was assessed through two different evaluation metrics, namely the area under the curve (AUC) of the receiver operating characteristic curve (Phillips, Anderson & Schapire, 2006) and the True Skill Statistics (TSS) (Allouche, Tsoar & Kadmon, 2006), this latter also providing information on model calibration (Jiménez-Valverde et al., 2013), with the 80% of the initial dataset used to build the models, and the remaining 20% used for the validation. For each of the 10 sets of pseudo-absences and each of the four modeling algorithms chosen, five iterations were performed, so that 200 models were finally generated. The EMs were built considering only the single ENMs exceeding both the thresholds, TSS > 0.8 and AUC > 0.7. The algorithms used to build the EMs were the “weighted mean of probabilities” (wmean), which permits to average the single models by weighting them on the basis of their AUC or TSS scores, the “median of probabilities” (median), and the “coefficient of variation of probabilities” (cv), which permits to map discrepancies among the single ENMs used to generate the EM (Thuiller et al., 2016). Moreover, the contribution of each predictor within the EMs was assessed by means of the algorithm-independent randomization procedure implemented in biomod2 (Thuiller et al., 2009; Bucklin et al., 2015). Special consideration was given to model extrapolation (i.e., environmental conditions within the projection scenarios falling outside the range of environmental conditions used to calibrate the models, see Elith & Leathwick (2009) for further details), quantifying it through the Multivariate Environmental Surface Similarity (Elith, Kearney & Phillips, 2010), computed through the function “mess” of the “dismo” package (Hijmans & Elith, 2016) in R (R Core Team, 2016). The degree of extrapolation calculated for each *GCM* × *RCP* × *year* combination was included in the modeling framework by processing the EMs’ projections to each year × RCP scenario through the MEDI algorithm, a form of weighted average which down-weights extrapolation (Iannella, Cerasoli & Biondi, 2017).

The gain, stability or loss of predicted suitable areas were calculated for each year × RCP scenario through the “BIOMOD_RangeSize” algorithm. Since this process needs binarized (i.e., presence/absence) maps, a binarization threshold was calculated through the “ecospat” R package (Di Cola et al., 2017), computing the threshold which maximizes the TSS (TSS-max) for each of the single ENMs selected to build the EMs and then averaging the different thresholds found. This procedure is particularly reliable when dealing with presence-background models, as it results in the same value of threshold that would be calculated from presence-absence models (Liu, White & Newell, 2013). This threshold was also compared to the values of the EM predictions for the current scenario read on the pseudo-absences points generated during model building. This permitted us to assess the proportion of pseudo-absences corresponding to false positives in the TSS-based binarized EM for current climatic conditions. Binarization of maps was performed through the “Reclassify” tool in ArcMap 10.0 (Esri, Redlands, CA, USA).

### Gap analysis

A gap analysis was performed in ArcMap 10.0 (Esri, Redlands, CA, USA) to assess *E. trinacris*’ current and future status of protection, evaluating the overlap between the PAs network and two different sets of target species’ data. First, presence points falling within the PAs were considered to assess the protection status of existing populations; then, EMs’ outcomes for both current and future scenarios were intersected with existing PAs, and the intersection extents were calculated. The shapefile of the PAs’ network was downloaded from the geo-portal of the Italian Ministry of the Environment (http://www.pcn.minambiente.it); it includes both nationally- (e.g., National parks and Reserves) and internationally- (e.g., Natura 2000 and Ramsar sites) established PAs. All marine PAs were excluded from the analyses.

Statistical analyses and graphics were performed using the package NCSS version 11 for Windows.

## Results

The spatial thinning process resulted in the selection of 36 out of the initial 39 localities. Presence records showed no significant spatial correlation, with a Moran’s *I* = −0.021 (expected value = −0.027), *z*-score = 0.147 and *p* = 0.883, confirming a random distribution pattern of the occurrence data. Nine bioclimatic variables (BIO3, BIO4, BIO7, BIO11, BIO13, BIO16, BIO17, BIO18 and BIO19) were selected as predictors for their low pairwise Pearson *r* coefficients; the correlation matrix used to choose these variables and the descriptive statistics for each of them are reported in Supplement 3.

Twenty-five models out of 200 exceeded both the TSS and AUC thresholds chosen, and were then selected as candidates for the ensemble modeling. The wmean EM showed high discrimination performance, with TSS = 0.885 and AUC = 0.972. Moreover, the high evaluation scores (TSS = 0.867 and AUC = 0.969) of the EM obtained through the median algorithm, which is less sensitive to outliers than the mean (Thuiller et al., 2016), and the apparent similarity of the median prediction maps with respect to the wmean ones, strengthen the results obtained from the whole ensemble modeling process.

The habitat suitability map for the current scenario, resulting from the wmean EM, shows a marked separation between suitable and non-suitable areas, with few areas at medium suitability (Fig. 1B). The reliability of these predictions is further confirmed by the cv map, which shows a low degree of EM uncertainty across the entire study area (Supplement 4).

**Figure 2:**
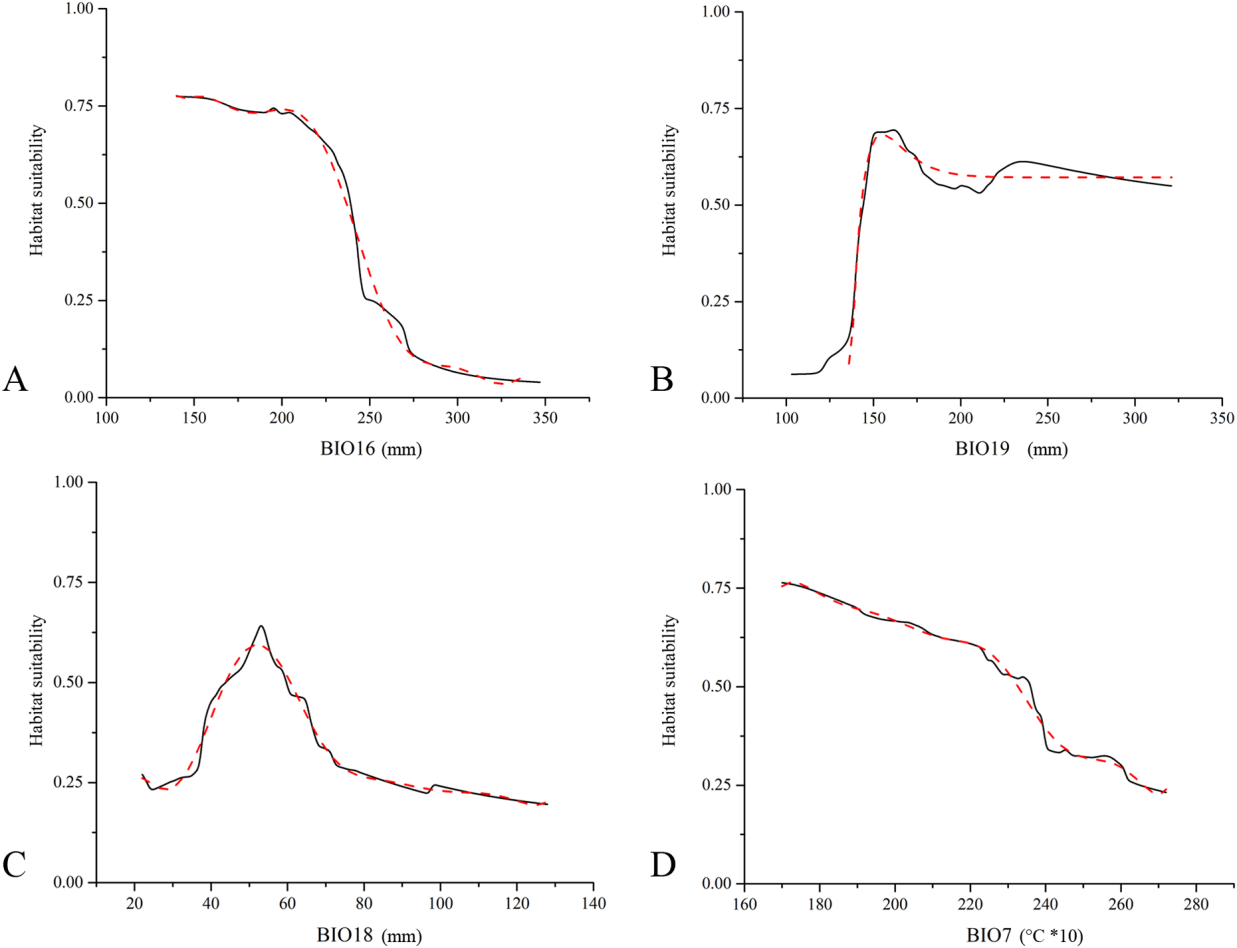
Marginal response curves obtained for *Emys trinacris.* Marginal response curves obtained for the four highest contributing predictors ((A) BIO16, Precipitation of Wettest Quarter; (B) BIO19, Precipitation of Coldest Quarter; (C) BIO18, Precipitation of Warmest Quarter; (D) BIO7, Temperature Annual Range) for *Emys trinacris* within the Ensemble Models built for the current bioclimatic conditions (solid line). For each response curve, the corresponding B-spline smoothed curve is reported with a red dashed line (*R*^2^ above 0.98 for all the four curves).

The assessment of the contribution for each predictor within the EMs resulted in a clear predominance of precipitation-linked bioclimatic variables. The amount of precipitation in the wettest quarter (BIO16), coldest quarter (BIO19) and warmest quarter (BIO18) represent the first, second and third most contributing variables, with 31.2%, 23.0% and 14.6% of the total contribution, respectively. The only temperature-related variable is temperature annual range (BIO7), with 9.6% of the total contribution. Response curves obtained for these four variables (Fig. 2) show that low values of BIO16 positively influence *E. trinacris*’ habitat suitability, while for BIO19 the peak in the predicted suitability corresponds to a small range of values surrounding 150 mm. Further, an increase of temperature annual range lowers the suitability for the target species (Fig. 2).

**Figure 3:**
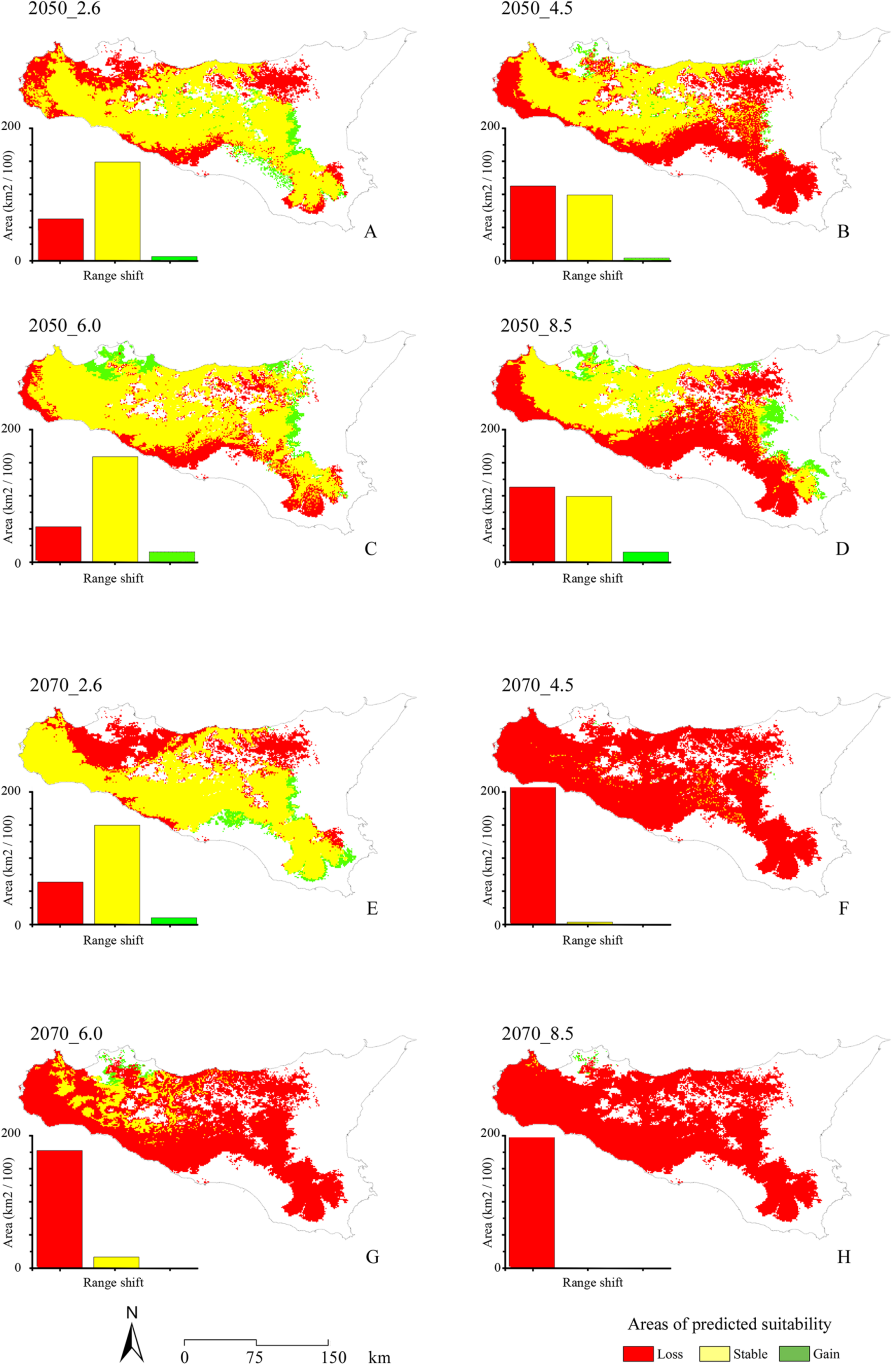
Range shifts for future projected scenarios. Maps of the range shifts resulting from the eight different future projected scenarios: 2.6, 4.5, 6.0 and 8.5 RCPs for 2050 (A, B, C and D, respectively) and 2070 (E, F, G and H, respectively). Area lost by the target species is reported in red, stable areas are reported in yellow, while areas gained is reported in green.

With respect to the modeled future projections, the variations of predicted suitable areas within the eight year × RCP scenarios are reported in Fig. 3; the discretization of the continuous maps resulting from the ensemble forecasting was carried out with a TSS maximization threshold = 0.604, which may be considered as very restrictive. The proportion of pseudo-absences which were assigned a habitat suitability value greater than the found TSS-max threshold is 11.5%, suggesting that a low number of pseudo-absences may be considered as potential false positives (i.e., good discriminative model performance).

A general and extensive loss of areas predicted as suitable under current climate is observable throughout the study area for all the future scenarios, coupled with no relevant increase in suitability (i.e., Gain) in other territories. In the four different 2050 RCPs scenarios, area loss goes along with the increase of radiative forcing, with an exception for the 6.0 scenario. All the 2070 RCPs scenarios show a loss of suitable areas proportional to the radiative forcing increase, including in this case also the 6.0 scenario.

**Figure 4:**
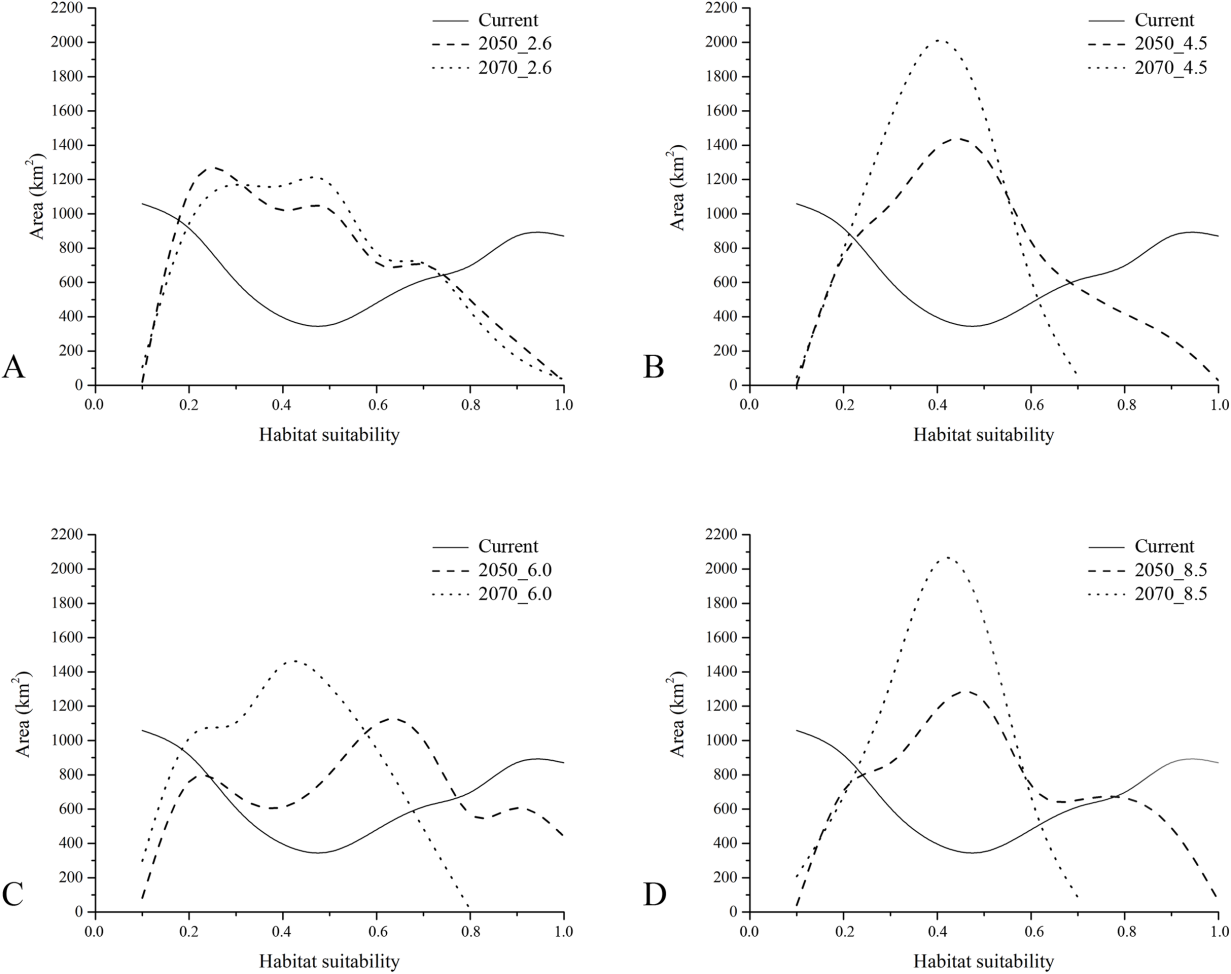
Results of gap analysis performed on Protected Areas and current—future habitat suitability. Areas (in km^2^) falling within Protected Areas, as resulting from the gap analysis performed on the raster maps of modelled habitat suitability for current (continuous), 2050 (dashed) and 2070 (dotted) under the four RCP scenarios considered (A = 2.6, B = 4.5, C = 6.0 and D = 8.5).

**Figure 5:**
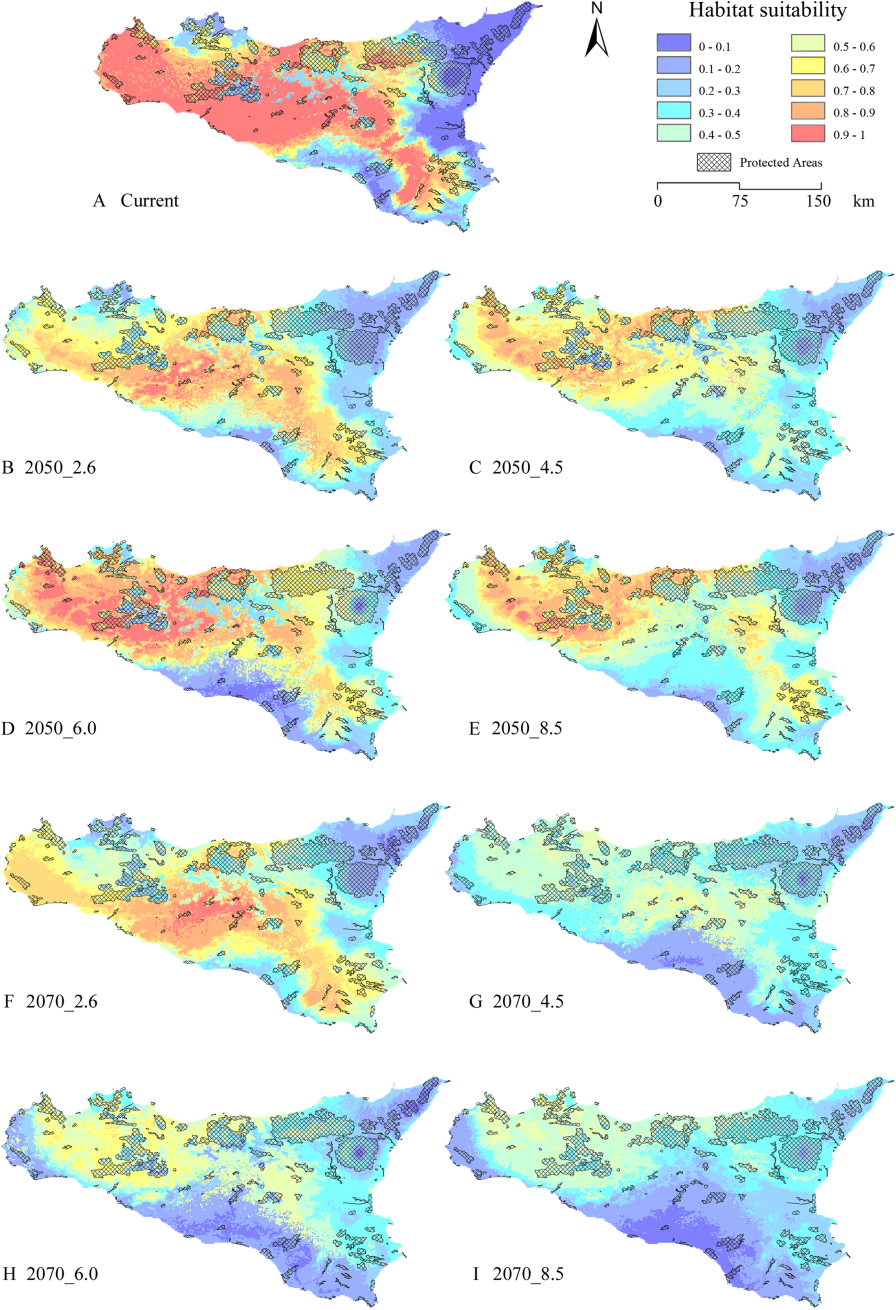
Protected Areas network and current—future habitat suitability maps. Maps reporting the overlay between modelled habitat suitability (low to high, in blue to red scale), for current ((A) and future scenarios (B) 2050_RCP2.6; (C) 2050_RCP4.5; (D) 2050_RCP6.0; (E) 2050_RCP8.5; (F) 2070_RCP2.6; (G) 2070_RCP4.5; (H) 2070_RCP6.0; (I) 2070_RCP8.5), and the current Protected Areas network (crosshatch pattern).

The gap analysis performed on the PAs’ network and *E. trinacris*’ presence sites resulted in 18 out of 39 records falling into PAs; this means that there are more than a half of the localities inhabited by the target species that are not covered by any form of legal protection. This trend pairs with the one emerging from the curves obtained through the gap analysis performed on the modelled habitat suitability for the current scenario (Fig. 4). In fact, approximately half of the PAs’ extent corresponds to areas predicted to be highly suitable (habitat suitability > 0.8) for *E. trinacris*, while the other protected half shows very low suitability (<0.2). With respect to the modelled future scenarios, PAs are predicted to mainly preserve areas with low-to-medium suitability (habitat suitability between 0.2 and 0.5) in all scenarios except the 2050_6.0 one, which shows a higher proportion of protected extent corresponding to a medium-to-high habitat suitability (Fig. 4C). The 4.5 and 8.5 RCPs show for both 2050 and 2070 a marked peak in the protection of areas whose suitability ranges from 0.4 to 0.5 (Figs. 4B and 4D). Overall, the 2050 scenarios show a higher degree of protection for areas with higher predicted suitability, if compared to the curves obtained for the 2070 RCPs. Moreover, Fig. 5 shows that a high number (and extent) of PAs is located in the eastern part of the island, which is predicted to host low suitable areas for the target species in both the current and the future scenarios.

## Discussion

The localities where *E. trinacris* is currently recorded are spread throughout the study area. Accordingly, the EM built on the current climatic conditions resulted in areas with high habitat suitability predicted to cover different parts of the island, not limited to the neighbourhoods of the presence sites (Fig. 1B). An apparent distinction is observable between suitable and non-suitable areas, with these latter mostly located in the eastern and southern portions of the study area, as well as across the main mountainous chains. This latter result agrees with the altitudinal limits of the target species, which is found from 0 to 1,007 m a.s.l. (Marrone et al., 2016). Moreover, the fact that a relatively wide area predicted to be highly suitable in the central and northern part of the island do not host recorded presence localities suggests that the potential distribution of the species might be wider than the current status of knowledge. Also, Turrisi (2008) reports the use of different aquatic environments in the northern (forest and artificial aquatic habitats) and western populations (coastal wetlands), while the central, eastern and southern populations live in mountain lakes and ponds, slow-moving river bends and residual wetlands, respectively. This habitat use suggests, considering the modelled habitat suitability, a very fragmented scenario, in which *E. trinacris*’ movements may be hindered by missing connections among these different habitats; this phenomenon could be even more marked considering *E. trinacris*’ low-dispersal capacity (Lo Valvo, D’Angelo & Regina, 2008; Lo Valvo et al., 2014), Sicily’s topographic heterogeneity and the reduction of past suitable environments due to land use change (Turrisi, 2008).

The response curves obtained for the four most contributing variables suggest that a certain range of precipitation positively influence *E. trinacris*’ modeled habitat suitability. In particular, the almost unimodal response trends to BIO16 and BIO19 can be interpreted taking into account the peculiarities of Mediterranean islands’ climate with respect to the habitats used by the target species during its entire life cycle, such as wetlands or slow-moving waterbodies. On one hand, the high predicted suitability corresponding to relatively low values of BIO16 is coherent with the environmental requirements of a species which is well acclimated to a Mediterranean island as Sicily, for which Cannarozzo, Noto & Viola (2006) showed historical negative trends in precipitation. These trends were observed especially in western and south-western parts of the island, corresponding to the greatest portion of the area predicted as highly suitable in the EMs obtained for current climate (Fig. 1B). On the other hand, the low predicted suitability for *E. trinacris* corresponding to low values of BIO19 well reflects the negative effect of decreasing winter precipitation, due to ongoing climate change, on different drivers of water availability, such as runoff and aquifers’ recharge, particularly pronounced in several Mediterranean islands (Lorenzo-Lacruz, Garcia & Moran-Tejeda, 2017; Montaldo & Sarigu, 2017).

Cycles of inundation heavily influence plant species composition in wetlands (Foti et al., 2012); considering that an appreciable portion of *E. trinacris*’ diet is made of voluntarily ingested aquatic plants (Ottonello et al., 2017), it can be assumed that precipitation exerts a heavy control on the target species. In addition, the other consistent part of the diet is made of aquatic invertebrates, which are the main organisms responsible for the degradation of plant matter; invertebrate communities and degradation processes are particularly dependent on hydroperiod, (Brooks, 2000; Battle & Golladay, 2001; Vanschoenwinkel et al., 2010), which, again, indirectly influences both the habitat and the diet of *E. trinacris*.

The sharp decrease of areas predicted as suitable for *E. trinacris’* in the projected EMs, for each of the eight year × RCP scenarios, further suggests a strong influence of precipitation patterns on the species’ environmental requirements; indeed, current and future changes in precipitation regimes in the Mediterranean region, with particular stress to the strong reduction of winter precipitation, has been evidenced in previous studies (Giorgi & Lionello, 2008; Barcikowska, Kapnick & Feser, 2017; Raymond et al., 2017), mainly connected to modifications in the North Atlantic Oscillation and Eastern Atlantic Pattern (Barcikowska, Kapnick & Feser, 2017).

The gap analysis performed on the predicted habitat suitability under current climate corroborates the lack of adequate protection for *E. trinacris* which already emerged from the assessment of the current protection status on the presence records: about a half of PAs covers highly suitable areas, while the other half covers area with poor predicted suitability. Furthermore, consulting the PAs’ technical sheets it emerged that 3 out of the 18 PAs in which the target species has been recorded do not even indicate in their plans *E. trinacris* as present within their borders. Overall, 17 management plans report the status of “Data Deficient” for *E. trinacris*’ local populations. The relevant data concerning the mentioned PAs are reported in Supplement 5, with the respective source web link provided.

Finally, results from the gap analysis performed considering the predicted suitability within the different future scenarios also show a low effectiveness of existing PAs in protecting the target species with respect to changing climate conditions. Indeed, from Fig. 4 it emerges that the peak of extent in territories protected by PAs falls in the low-to-medium predicted suitability range (0.2–0.5) under all the RCP scenarios, thus demonstrating the ongoing and forthcoming problems for the conservation of this endemic species.

## Conclusions

Stability in precipitation amount and temperature variations strongly affects *E. trinacris’* suitable habitats. Considering the high contribution values of precipitation-related variables, the water balance of the aquatic sites inhabited by this species resulted to be of primary importance for its conservation, which is jeopardized by the ongoing process of climate change. Considering the RCP 2.6 within the modeling framework, even though it represents a no more plausible scenario of future radiative forcing, gave us the opportunity to investigate how the potential distribution of a species acclimated to the current Mediterranean environments could response to moderate warming. In fact, the noticeable stability of predicted suitable areas resulting for both 2050 and 2070 under this RCP is in opposition to the outcomes from the projections under the RCP 8.5, which instead reported high rates of loss of suitable areas. Thus, these two “boundary” RCPs might give important information about the responses of projected ENMs to diametrically opposed GHG emissions trajectories, and should be taken into consideration when modeling species’ distribution in relation to climate change. On the other side, the gaps in the PAs’ regional network revealed a critical situation for the conservation of *E. trinacris*, showing the adversities that existing PAs will have to face in protecting both current presence localities and future suitable areas, with these latter which may be used as refuge areas. The highlighted management shortfall, coupled with the forecasts of future extreme meteorological events within the Mediterranean basin, clearly demonstrates the weakness of the current conservation status of this threatened endemic species. Therefore, PAs should actively look for adequate solutions to preserve the populations falling within their boundaries, such as direct (e.g., population monitoring) and indirect (e.g., water bodies management) conservation practices. Further, PAs should encourage field research activities, in order to improve the knowledge about the autoecological features of the species and look for possible disturbances (e.g., invasive alien species). With respect to *E. trinacris*’ populations (and areas with high suitability) outside PAs, local managers and stakeholders should take into consideration the possibility of ad-hoc conservation measures.

## Supplemental Information

10.7717/peerj.4969/supp-1 Supplemental Information 1 Coordinates of* Emys trinacris’* presence points and respective sources. The coordinates of *Emys trinacris’* presence points (WGS84 reference system) are provided at lower resolution than the one used to build the Ensemble Models in order to avoid the risk of illegal withdrawal by poachers, who could use published data to collect individuals from occurrence localities.
Sources: ^1^Ruffo, S., & Stoch, F. (Eds.). (2005). Checklist e distribuzione della fauna italiana: 10.000 specie terrestri e delle acque interne. Museo civico di storia naturale di Verona. ^2^iNaturalist.org web application at http://www.inaturalist.org (accessed 10 November 2017). ^3^SCI technical sheets found at http://www.artasicilia.eu/old_site/web/natura2000/ (accessed 8 November 2017).
All other sources are reported in the main text References.

10.7717/peerj.4969/supp-2 Supplemental Information 2 The set of the nineteen bioclimatic variables considered as candidate predictors (from Worldclim.org), with their codes and explication.

10.7717/peerj.4969/supp-3 Supplemental Information 3 Correlation matrix of the 19 candidate predictors and descriptive statistics of the ones selected for model building. Above, the correlation matrix built among the 19 candidate predictors. Variables showing a Pearson correlation | r | > 0.85, discarded from model building, are highlighted in yellow. Below, a table reporting the descriptive statistics (Mean = mean value of the predictor; SD = standard deviation; Min. Value = minimum value of the predictor; Max. Value = maximum value of the predictor) for the bioclimatic variables chosen as predictors.

10.7717/peerj.4969/supp-4 Supplemental Information 4 Coefficient of variation map. Coefficient of variation (cv) map resulting from the Ensemble Modelling process performed over *Emys trinacris*’ records dataset.

10.7717/peerj.4969/supp-5 Supplemental Information 5 Subset of Natura 2000 sites involved in the gap analysis. The sites listed below are the ones not reporting *Emys trinacris* within their technical sheets (Status = NO) (even though the species is found within their respective borders) or defining a “Data Deficient” status (DD). Sites of Community Importance (Habitats Directive) are reported as “SCI” and Special Protection Area (Birds Directive) are reported as “SPA” in Protected Areas’ designation type (Design). The field “Site name” reports the original PAs’ names; the “Status_yr” reports the year of PAs’ first proposal as SCI or SPA; “Web source” reports the link to the national official repository (the ftp web link to the Italian Ministry of the Environment) for the technical sheets corresponding to each site.

## Additional Information and Declarations

### Data Availability

The following information was supplied regarding data availability: The coordinates of *Emys trinacris*’ presence points used to build the Ensemble Models and to perform the gap analysis are provided as a Supplemental File.
