# Supplementary material for: Coupling GIS spatial analysis and Ensemble Niche Modelling to investigate climate change-related threats to the Sicilian pond turtle Emys trinacris, an endangered species from the Mediterranean
Source: PeerJ. 2018 Jun 5;6:e4969. doi: 10.7717/peerj.4969 (PMC5993018; doi:10.7717/peerj.4969)
Supplement: Supplemental Information 1 — The coordinates of Emys trinacris’ presence points (WGS84 reference system) are provided at lower resolution than the one used to build the Ensemble Models in order to avoid the risk of illegal withdrawal by poachers, who could use published data to collect individuals from occurrence localities. Sources: 1Ruffo, S., & Stoch, F. (Eds.). (2005). Checklist e distribuzione della fauna italiana: 10.000 specie terrestri e delle acque interne. Museo civico di storia naturale di Verona. 2iNaturalist.org web application at http://www.inaturalist.org (accessed 10 November 2017). 3SCI technical sheets found at http://www.artasicilia.eu/old_site/web/natura2000/ (accessed 8 November 2017). All other sources are reported in the main text References. [file peerj-06-4969-s001.docx]

**Supplemental information**

**Supplement 1. Supporting dataset. Coordinates of *Emys trinacris’* presence points**

The coordinates of *Emys trinacris’* presence points (WGS84 reference system) are provided at lower resolution than the one used to build the Ensemble Models in order to avoid the risk of illegal withdrawal by poachers, who could use published data to collect individuals from occurrence localities. Sources:

^1^ Ruffo, S., & Stoch, F. (Eds.). (2005). Checklist e distribuzione della fauna italiana: 10.000 specie terrestri e delle acque interne. Museo civico di storia naturale di Verona.

^2^ iNaturalist.org web application at http://www.inaturalist.org (accessed 10 November 2017).

^3^ SCI technical sheets found at http://www.artasicilia.eu/old_site/web/natura2000/ (accessed 8 November 2017).

All other sources are reported in the main text References.

| **Species name** | **X** | **Y** | **Data source** |
| --- | --- | --- | --- |
| *Emys trinacris* | 14.81 | 38.09 | CKmap^1^ |
| *Emys trinacris* | 14.39 | 37.88 | CKmap^1^ |
| *Emys trinacris* | 14.78 | 37.82 | CKmap^1^ |
| *Emys trinacris* | 14.34 | 37.02 | CKmap^1^ |
| *Emys trinacris* | 12.65 | 37.61 | D'Angelo et al. 2008 |
| *Emys trinacris* | 13.33 | 37.37 | D'Angelo et al. 2008 |
| *Emys trinacris* | 13.53 | 37.75 | iNaturalist^2^ |
| *Emys trinacris* | 13.41 | 37.73 | iNaturalist^2^ |
| *Emys trinacris* | 14.56 | 37.84 | Natura 2000^3^ |
| *Emys trinacris* | 14.39 | 37.83 | Natura 2000^3^ |
| *Emys trinacris* | 14.81 | 37.65 | Natura 2000^3^ |
| *Emys trinacris* | 14.80 | 37.72 | Natura 2000^3^ |
| *Emys trinacris* | 14.56 | 37.44 | Natura 2000^3^ |
| *Emys trinacris* | 14.07 | 37.39 | Natura 2000^3^ |
| *Emys trinacris* | 14.40 | 37.90 | Personal data (M. Iannella) |
| *Emys trinacris* | 14.67 | 37.94 | Personal data (M. Iannella) |
| *Emys trinacris* | 13.29 | 37.85 | Personal data (M. Iannella) |
| *Emys trinacris* | 14.31 | 37.51 | Personal data (M. Iannella) |
| *Emys trinacris* | 13.98 | 37.31 | Personal data (M. Iannella) |
| *Emys trinacris* | 12.89 | 38.02 | Vamberger et al. 2015 |
| *Emys trinacris* | 14.37 | 37.93 | Vamberger et al. 2015 |
| *Emys trinacris* | 14.58 | 37.90 | Vamberger et al. 2015 |
| *Emys trinacris* | 12.96 | 37.99 | Vamberger et al. 2015 |
| *Emys trinacris* | 14.40 | 37.75 | Vamberger et al. 2015 |
| *Emys trinacris* | 13.30 | 37.81 | Vamberger et al. 2015 |
| *Emys trinacris* | 12.80 | 37.82 | Vamberger et al. 2015 |
| *Emys trinacris* | 12.44 | 37.80 | Vamberger et al. 2015 |
| *Emys trinacris* | 13.40 | 37.75 | Vamberger et al. 2015 |
| *Emys trinacris* | 12.59 | 37.65 | Vamberger et al. 2015 |
| *Emys trinacris* | 12.79 | 37.68 | Vamberger et al. 2015 |
| *Emys trinacris* | 13.08 | 37.51 | Vamberger et al. 2015 |
| *Emys trinacris* | 15.10 | 37.51 | Vamberger et al. 2015 |
| *Emys trinacris* | 13.20 | 37.47 | Vamberger et al. 2015 |
| *Emys trinacris* | 13.42 | 37.33 | Vamberger et al. 2015 |
| *Emys trinacris* | 13.58 | 37.31 | Vamberger et al. 2015 |
| *Emys trinacris* | 13.94 | 37.11 | Vamberger et al. 2015 |
| *Emys trinacris* | 15.04 | 37.16 | Vamberger et al. 2015 |
| *Emys trinacris* | 14.73 | 36.93 | Vamberger et al. 2015 |
| *Emys trinacris* | 15.10 | 36.81 | Vamberger et al. 2015 |
