# Supplementary material for: Coupling GIS spatial analysis and Ensemble Niche Modelling to investigate climate change-related threats to the Sicilian pond turtle Emys trinacris, an endangered species from the Mediterranean
Source: PeerJ. 2018 Jun 5;6:e4969. doi: 10.7717/peerj.4969 (PMC5993018; doi:10.7717/peerj.4969)
Supplement: Supplemental Information 2 [file peerj-06-4969-s002.docx]

**Supplemental information**

**Supplement 2.** The set of the nineteen bioclimatic variables considered as candidate predictors (from Worldclim.org), with their codes and explication.

BIO1 = Annual Mean Temperature

BIO2 = Mean Diurnal Range (Mean of monthly (max temp – min temp))

BIO3 = Isothermality (BIO2/BIO7)*100

BIO4 = Temperature Seasonality (standard deviation*100)

BIO5 = Max Temperature of Warmest Month

BIO6 = Min Temperature of Coldest Month

BIO7 = Temperature Annual Range (BIO5-BIO6)

BIO8 = Mean Temperature of Wettest Quarter

BIO9 = Mean Temperature of Driest Quarter

BIO10 = Mean Temperature of Warmest Quarter

BIO11 = Mean Temperature of Coldest Quarter

BIO12 = Annual Precipitation

BIO13 = Precipitation of Wettest Month

BIO14 = Precipitation of Driest Month

BIO15 = Precipitation Seasonality (Coefficient of Variation)

BIO16 = Precipitation of Wettest Quarter

BIO17 = Precipitation of Driest Quarter

BIO18 = Precipitation of Warmest Quarter

BIO19 = Precipitation of Coldest Quarter
