# Supplementary material for: Coupling GIS spatial analysis and Ensemble Niche Modelling to investigate climate change-related threats to the Sicilian pond turtle Emys trinacris, an endangered species from the Mediterranean
Source: PeerJ. 2018 Jun 5;6:e4969. doi: 10.7717/peerj.4969 (PMC5993018; doi:10.7717/peerj.4969)
Supplement: Supplemental Information 3 — Above, the correlation matrix built among the 19 candidate predictors. Variables showing a Pearson correlation | r | > 0.85, discarded from model building, are highlighted in yellow. Below, a table reporting the descriptive statistics (Mean = mean value of the predictor; SD = standard deviation; Min. Value = minimum value of the predictor; Max. Value = maximum value of the predictor) for the bioclimatic variables chosen as predictors. [file peerj-06-4969-s003.docx]

**Supplemental information**

**Supplement 3.** **Correlation matrix of the 19 candidate predictors and descriptive statistics of the ones selected for model building.**

Above, the correlation matrix built among the 19 candidate predictors. Variables showing a Pearson correlation | r | > 0.85, discarded from model building, are highlighted in yellow. Below, a table reporting the descriptive statistics (Mean = mean value of the predictor; SD = standard deviation; Min. Value = minimum value of the predictor; Max. Value = maximum value of the predictor) for the bioclimatic variables chosen as predictors.

|  | | **BIO1** | | **BIO2** | | **BIO3** | | **BIO4** | | **BIO5** | | **BIO6** | | **BIO7** | | **BIO8** | | **BIO9** | | **BIO10** | | **BIO11** | | **BIO12** | | **BIO13** | | **BIO14** | | **BIO15** | | **BIO16** | | **BIO17** | | **BIO18** | | | **BIO19** | | |  |
| --- | --- | --- | --- | --- | --- | --- | --- | --- | --- | --- | --- | --- | --- | --- | --- | --- | --- | --- | --- | --- | --- | --- | --- | --- | --- | --- | --- | --- | --- | --- | --- | --- | --- | --- | --- | --- | --- | --- | --- | --- | --- | --- |
| **BIO1** | 1.00 | |  | |  | |  | |  | |  | |  | |  | |  | |  | |  | |  | |  | |  | |  |  |  | |  | |  | |  | | |  |  |  |
| **BIO2** | 0.42 | | 1.00 | |  | |  | |  | |  | |  | |  | |  | |  | |  | |  | |  | |  | |  |  |  | |  | |  | |  | | |  |  |  |
| **BIO3** | 0.74 | | 0.87 | | 1.00 | |  | |  | |  | |  | |  | |  | |  | |  | |  | |  | |  | |  |  |  | |  | |  | |  | | |  |  |  |
| **BIO4** | -0.86 | | -0.19 | | -0.64 | | 1.00 | |  | |  | |  | |  | |  | |  | |  | |  | |  | |  | |  |  |  | |  | |  | |  | | |  |  |  |
| **BIO5** | 0.89 | | 0.71 | | 0.83 | | -0.59 | | 1.00 | |  | |  | |  | |  | |  | |  | |  | |  | |  | |  |  |  | |  | |  | |  | | |  |  |  |
| **BIO6** | 0.97 | | 0.19 | | 0.59 | | -0.91 | | 0.77 | | 1.00 | |  | |  | |  | |  | |  | |  | |  | |  | |  |  |  | |  | |  | |  | | |  |  |  |
| **BIO7** | -0.41 | | 0.57 | | 0.10 | | 0.69 | | 0.03 | | -0.62 | | 1.00 | |  | |  | |  | |  | |  | |  | |  | |  |  |  | |  | |  | |  | | |  |  |  |
| **BIO8** | 0.98 | | 0.40 | | 0.74 | | -0.88 | | 0.86 | | 0.95 | | -0.43 | | 1.00 | |  | |  | |  | |  | |  | |  | |  |  |  | |  | |  | |  | | |  |  |  |
| **BIO9** | 0.97 | | 0.45 | | 0.70 | | -0.73 | | 0.94 | | 0.91 | | -0.28 | | 0.94 | | 1.00 | |  | |  | |  | |  | |  | |  |  |  | |  | |  | |  | | |  |  |  |
| **BIO10** | 0.99 | | 0.47 | | 0.73 | | -0.77 | | 0.94 | | 0.93 | | -0.30 | | 0.96 | | 0.99 | | 1.00 | |  | |  | |  | |  | |  |  |  | |  | |  | |  | | |  |  |  |
| **BIO11** | 1.00 | | 0.39 | | 0.74 | | -0.90 | | 0.86 | | 0.98 | | -0.47 | | 0.98 | | 0.95 | | 0.97 | | 1.00 | |  | |  | |  | |  |  |  | |  | |  | |  | | |  |  |  |
| **BIO12** | -0.34 | | -0.14 | | -0.33 | | 0.44 | | -0.21 | | -0.34 | | 0.27 | | -0.41 | | -0.26 | | -0.28 | | -0.36 | | 1.00 | |  | |  | |  |  |  | |  | |  | |  | | |  |  |  |
| **BIO13** | 0.06 | | 0.63 | | 0.46 | | 0.08 | | 0.31 | | -0.10 | | 0.53 | | 0.02 | | 0.09 | | 0.11 | | 0.04 | | 0.51 | | 1.00 | |  | |  |  |  | |  | |  | |  | | |  |  |  |
| **BIO14** | -0.76 | | -0.30 | | -0.61 | | 0.79 | | -0.60 | | -0.76 | | 0.44 | | -0.80 | | -0.68 | | -0.70 | | -0.78 | | 0.77 | | 0.25 | | 1.00 | |  |  |  | |  | |  | |  | | |  |  |  |
| **BIO15** | 0.72 | | 0.61 | | 0.86 | | -0.73 | | 0.66 | | 0.63 | | -0.17 | | 0.74 | | 0.62 | | 0.67 | | 0.73 | | -0.57 | | 0.27 | | -0.82 | | 1.00 |  |  | |  | |  | |  | | |  |  |  |
| **BIO16** | -0.07 | | 0.17 | | 0.03 | | 0.20 | | 0.08 | | -0.12 | | 0.29 | | -0.14 | | -0.02 | | -0.02 | | -0.09 | | 0.92 | | 0.77 | | 0.54 | | -0.21 | 1.00 |  | |  | |  | |  | | |  |  |  |
| **BIO17** | -0.74 | | -0.27 | | -0.58 | | 0.75 | | -0.59 | | -0.74 | | 0.43 | | -0.78 | | -0.67 | | -0.69 | | -0.76 | | 0.84 | | 0.34 | | 0.98 | | -0.78 | 0.65 | 1.00 | |  | |  | |  | | |  |  |  |
| **BIO18** | -0.17 | | -0.02 | | -0.17 | | 0.29 | | -0.06 | | -0.19 | | 0.22 | | -0.24 | | -0.10 | | -0.12 | | -0.20 | | 0.81 | | 0.48 | | 0.65 | | -0.45 | 0.76 | 0.69 | | 1.00 | |  | |  | | |  |  |  |
| **BIO19** | -0.32 | | -0.06 | | -0.30 | | 0.49 | | -0.13 | | -0.34 | | 0.37 | | -0.39 | | -0.21 | | -0.24 | | -0.35 | | 0.93 | | 0.46 | | 0.72 | | -0.55 | 0.85 | 0.77 | | 0.73 | | 1.00 | | |  | | |  |  |

|  | **Mean** | **SD** | **Min. Value** | **Max. Value** |
| --- | --- | --- | --- | --- |
| **BIO3** | 107.7 | 39.3 | 24.0 | 128.0 |
| **BIO4** | 5474.6 | 359.3 | 4507.0 | 6454.0 |
| **BIO7** | 224.1 | 13.0 | 170.0 | 273.0 |
| **BIO11** | 92.6 | 22.5 | -75.0 | 136.0 |
| **BIO13** | 84.5 | 11.7 | 52.0 | 120.0 |
| **BIO16** | 224.7 | 24.7 | 140.0 | 347.0 |
| **BIO17** | 28.8 | 11.0 | 6.0 | 128.0 |
| **BIO18** | 53.4 | 12.9 | 21.0 | 128.0 |
| **BIO19** | 176.5 | 30.8 | 103.0 | 321.0 |
