# Supplementary figures and images for: Coupling GIS spatial analysis and Ensemble Niche Modelling to investigate climate change-related threats to the Sicilian pond turtle Emys trinacris, an endangered species from the Mediterranean
Source: PeerJ. 2018 Jun 5;6:e4969. doi: 10.7717/peerj.4969 (PMC5993018; doi:10.7717/peerj.4969)

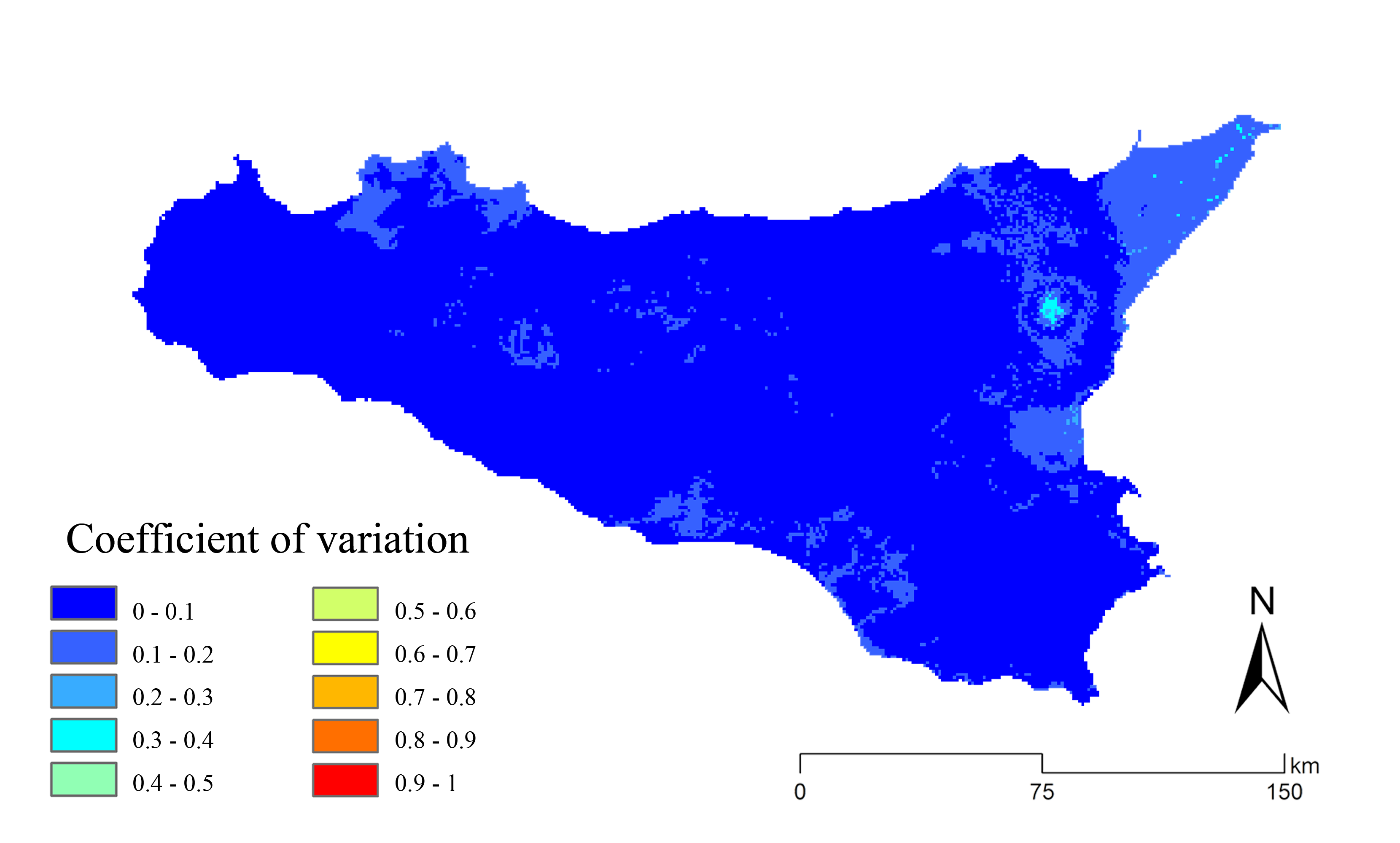

Supplement: Supplemental Information 4 — Coefficient of variation (cv) map resulting from the Ensemble Modelling process performed over Emys trinacris’ records dataset. [file peerj-06-4969-s004.png]
