# Supplementary material for: Coupling GIS spatial analysis and Ensemble Niche Modelling to investigate climate change-related threats to the Sicilian pond turtle Emys trinacris, an endangered species from the Mediterranean
Source: PeerJ. 2018 Jun 5;6:e4969. doi: 10.7717/peerj.4969 (PMC5993018; doi:10.7717/peerj.4969)
Supplement: Supplemental Information 5 — The sites listed below are the ones not reporting Emys trinacris within their technical sheets (Status = NO) (even though the species is found within their respective borders) or defining a “Data Deficient” status (DD). Sites of Community Importance (Habitats Directive) are reported as “SCI” and Special Protection Area (Birds Directive) are reported as “SPA” in Protected Areas’ designation type (Design). The field “Site name” reports the original PAs’ names; the “Status_yr” reports the year of PAs’ first proposal as SCI or SPA; “Web source” reports the link to the national official repository (the ftp web link to the Italian Ministry of the Environment) for the technical sheets corresponding to each site. [file peerj-06-4969-s005.docx]

**Supplemental information**

**Supplement 5.** **Supporting table. Subset of Natura 2000 sites involved in the gap analysis.** The sites listed below are the ones not reporting *Emys trinacris* within their technical sheets (Status = NO) (even though the species is found within their respective borders) or defining a “Data Deficient” status (DD). Sites of Community Importance (Habitats Directive) are reported as “SCI” and Special Protection Area (Birds Directive) are reported as “SPA” in Protected Areas’ designation type (Design). The field “Site name” reports the original PAs’ names; the “Status_yr” reports the year of PAs’ first proposal as SCI or SPA; “Web source” reports the link to the national official repository (the ftp web link to the Italian Ministry of the Environment) for the technical sheets corresponding to each site.

| **Site name** | **Status** | **Web source** | **Design** | **Status_yr** |
| --- | --- | --- | --- | --- |
| Rocca Busambra e Rocche di Rao | NO | ftp://ftp.minambiente.it/pnm/Natura2000/TrasmissioneCE_2015/schede_mappe/Sicilia/ZSC_schede/Site_ITA020008.pdf | SCI | 1995 |
| Foce del Fiume Verdura | NO | ftp://ftp.minambiente.it/pnm/Natura2000/TrasmissioneCE_2015/schede_mappe/Sicilia/ZSC_schede/Site_ITA040004.pdf | SCI | 1995 |
| Monte Pelato | NO | ftp://ftp.minambiente.it/pnm/Natura2000/TrasmissioneCE_2015/schede_mappe/Sicilia/SIC_schede/Site_ITA030039.pdf | SCI | 1995 |
| Monti Nebrodi | DD | ftp://ftp.minambiente.it/pnm/Natura2000/TrasmissioneCE_2015/schede_mappe/Sicilia/ZPS_schede/Site_ITA030043.pdf | SPA | 2005 |
| Serra del Re, Monte Soro e Biviere di Cesarò | DD | ftp://ftp.minambiente.it/pnm/Natura2000/TrasmissioneCE_2015/schede_mappe/Sicilia/SIC_schede/Site_ITA030038.pdf | SCI | 1995 |
| Lago di Pergusa | DD | ftp://ftp.minambiente.it/pnm/Natura2000/TrasmissioneCE_2015/schede_mappe/Sicilia/ZSC_schede/Site_ITA060002.pdf | SCI/SPA | 1995 |
| Contrada Caprara | DD | ftp://ftp.minambiente.it/pnm/Natura2000/TrasmissioneCE_2015/schede_mappe/Sicilia/ZSC_schede/Site_ITA060011.pdf | SCI | 1995 |
| Poggio S. Maria | DD | ftp://ftp.minambiente.it/pnm/Natura2000/TrasmissioneCE_2015/schede_mappe/Sicilia/ZSC_schede/Site_ITA070011.pdf | SCI | 1995 |
| Monti Sicani, Rocca Busambra e Bosco della Ficuzza | DD | ftp://ftp.minambiente.it/pnm/Natura2000/TrasmissioneCE_2015/schede_mappe/Sicilia/ZPS_schede/Site_ITA020048.pdf | SPA | 2005 |
| Laghetti di Preola e Gorghi Tondi, Sciare di Mazara e Pantano Leone | DD | ftp://ftp.minambiente.it/pnm/Natura2000/TrasmissioneCE_2015/schede_mappe/Sicilia/SIC_schede/Site_ITA010005.pdf | SPA | 2005 |
| Pantani della Sicilia sud-orientale, Morghella, di Marzamemi, di Punta Pilieri e Vendicari | DD | ftp://ftp.minambiente.it/pnm/Natura2000/TrasmissioneCE_2015/schede_mappe/Sicilia/ZPS_schede/Site_ITA090029.pdf | SPA | 2005 |
| Lago Ogliastro | DD | ftp://ftp.minambiente.it/pnm/Natura2000/TrasmissioneCE_2015/schede_mappe/Sicilia/ZSC_schede/Site_ITA060001.pdf | SCI | 1995 |
| Laghetti di Preola e Gorghi Tondi e Sciare di Mazara | DD | ftp://ftp.minambiente.it/pnm/Natura2000/TrasmissioneCE_2015/schede_mappe/Sicilia/SIC_schede/Site_ITA010005.pdf | SCI | 1995 |
| Foce del Magazzolo, Foce del Platani, Capo Bianco, Torre Salsa | DD | ftp://ftp.minambiente.it/pnm/Natura2000/TrasmissioneCE_2015/schede_mappe/Sicilia/SIC_schede/Site_ITA040003.pdf | SCI | 1995 |
| Monte Sambughetti, Monte Campanito | DD | ftp://ftp.minambiente.it/pnm/Natura2000/TrasmissioneCE_2015/schede_mappe/Sicilia/ZSC_schede/Site_ITA060006.pdf | SCI | 1995 |
| Forre laviche del Fiume Simeto | DD | ftp://ftp.minambiente.it/pnm/Natura2000/TrasmissioneCE_2015/schede_mappe/Sicilia/ZSC_schede/Site_ITA070026.pdf | SCI | 1995 |
| Lago di Ancipa | DD | ftp://ftp.minambiente.it/pnm/Natura2000/TrasmissioneCE_2015/schede_mappe/Sicilia/SIC_schede/Site_ITA060005.pdf | SCI | 1995 |
| Vallone Laccaretta e Urio Quattrocchi | DD | ftp://ftp.minambiente.it/pnm/Natura2000/TrasmissioneCE_2015/schede_mappe/Sicilia/SIC_schede/Site_ITA030017.pdf | SCI | 1995 |
| Biviere e Macconi di Gela | DD | ftp://ftp.minambiente.it/pnm/Natura2000/TrasmissioneCE_2015/schede_mappe/Sicilia/SIC_schede/Site_ITA050001.pdf | SCI | 1995 |
| Vendicari | DD | ftp://ftp.minambiente.it/pnm/Natura2000/TrasmissioneCE_2015/schede_mappe/Sicilia/SIC_schede/Site_ITA090002.pdf | SCI | 1995 |
